# A model to optimize public health care and downstage breast cancer in limited-resource populations in southern Brazil. (Porto Alegre Breast Health Intervention Cohort)

**DOI:** 10.1186/1471-2458-9-83

**Published:** 2009-03-13

**Authors:** Maira Caleffi, Rodrigo A Ribeiro, Dakir L Duarte Filho, Patrícia Ashton-Prolla, Ademar J Bedin, Giovana P Skonieski, Juliana M Zignani, Juliana Giacomazzi, Luciane R Franco, Márcia Graudenz, Paula Pohlmann, Jefferson G Fernandes, Philip Kivitz, Bernardete Weber

**Affiliations:** 1 Associação Hospitalar Moinhos de Vento, Porto Alegre, Brazil; 2 Department of Genetics, Universidade Federal do Rio Grande do Sul, Brazil; 3 Hospital de Clínicas de Porto Alegre, Brazil; 4 Post-Graduate Program in Genetics and Molecular Biology, Universidade Federal do Rio Grande do Sul, Brazil; 5 Post-Graduate Course in Medicine, Universidade Federal do Rio Grande do Sul, Brazil; 6 Secretaria Municipal de Saúde, Porto Alegre, Brazil; 7 Vanderbilt University, Nashville, USA; 8 Stanford University, San Francisco, USA; 9 Rua Ramiro Barcelos 910, 11º andar, CEP 90035-001, Porto Alegre, RS, Brazil

## Abstract

**Background:**

Breast cancer (BC) is a major public health problem, with rising incidence in many regions of the globe. Although mortality has recently dropped in developed countries, death rates are still increasing in some developing countries, as seen in Brazil. Among the reasons for this phenomenon are the lack of structured screening programs, a long waiting period between diagnosis and treatment, and lack of access to health services for a large proportion of the Brazilian population.

**Methods and design:**

Since 2004, an intervention study in a cohort of women in Southern Brazil, denominated Porto Alegre Breast Health Intervention Cohort, is being conducted in order to test the effectiveness and cost-effectiveness of a model for BC early detection and treatment. In this study, over 4,000 women from underserved communities aged 40 to 69 years are being screened annually with mammography and clinical breast examination performed by a multidisciplinary team, which also involves nutritional counseling and genetic cancer risk assessment. Risk factors for BC development are also being evaluated. Active search of participants by lay community health workers is one of the major features of our program. The accrual of new participants was concluded in 2006 and the study will last for 10 years. The main goal of the study is to demonstrate significant downstaging of BC in an underserved population through proper screening, attaining a higher rate of early-stage BC diagnoses than usually seen in women diagnosed in the Brazilian Public Health System. Preliminary results show a very high BC incidence in this population (117 cases per 100,000 women per year), despite a low prevalence of classical risk factors.

**Discussion:**

This study will allow us to test a model of BC early diagnosis and treatment and evaluate its cost-effectiveness in a developing country where the mortality associated with this disease is very high. Also, it might contribute to the evaluation of risk factors in a population with a different ethnic background from that studied in developed countries. If our model is proven effective, it may be replicated in other parts of the globe where BC is also a major public health problem.

## Background

### Breast cancer as a major public health problem

Breast cancer (BC) incidence rates have increased significantly worldwide, and today is the most common non-skin cancer in women in almost all parts of the globe. The incidence is higher in the United States and Northern Europe, intermediate in Southern and Eastern Europe and South America, and lower in Asia [1].

Although BC incidence rates are still increasing in developed countries, BC mortality rates have declined in recent years in these nations, what can be explained by greater breast cancer awareness, by the guarantee of health care access and by the adoption of public policies towards early tumor detection. In countries who have successfully implemented nationwide mammography screening programs, such as the United Kingdom [2], the Netherlands [3] and Sweden [4,5], the mortality rates have dropped by at least 20%.

In contrast, in Brazil and other developing countries, both mortality and incidence rates have steadily increased in recent years. For example, the age-standardized mortality (ASM) related to BC in Brazil has grown from 10.74 per 100,000 women in 2000 to 12.32 in 2005 [6], which probably reflects the delay in diagnosis: almost half of the BC cases in Brazil are diagnosed in stages III and IV [7-9]. When breast tumors in Brazil are analyzed according to their staging, mortality is similar to studies conducted in developed countries, supporting the idea that late diagnosis is one of the main causes of the high case fatality rate observed in the country [10].

The State of Rio Grande do Sul (RS, the southernmost state in the country) presents the second highest breast cancer incidence rate in the country, with an estimated rate of 85.50 new cases per 100,000 women in 2008 – a number comparable to the USA and North Europe. The State's capital, Porto Alegre, has an even higher BC incidence rate, with 119.72 new cases per 100,000 women projected for the current year [11].

### Breast cancer screening in Brazil

In Brazil, approximately 75% of the population has access to health care only through the Brazilian Public Health System (PHS) [12], which is therefore responsible for the provision of breast health care to the majority of the population. The initial health care is provisioned in primary care, by the basic healthcare units (BHUs), the first access to health provision in the country, since the referral for other levels of care is their attribution. In our city, Porto Alegre, there are 87 BHUs, for a total population of 1.45 million inhabitants. This infrastructure is one of the most organized primary care setting in the whole country.

In Brazil, the current national recommendation for breast cancer screening calls for mammography at two-year intervals, targeted at women between the ages of 50 and 69 years [13]. These recommendations are not in accordance with those published by the Brazilian Society of Breast Disease [14], which recommends annual screening between the ages of 40 and 69 years.

Although the government guideline containing these recommendations has been published in 2004, there is not actually a national screening program, since there is no provision of financing nor training of health care professionals capable to provide service to all eligible women between ages 50 and 69 years. In the areas of the city of Porto Alegre included in this project, there used to be no mammographic reference service. Women with breast symptoms used to be referred to the city central area, where they were offered the services of a breast specialist necessary for diagnostic procedures (mammography, ultrasound, core biopsy) and treatment, with very long waiting times for each step of the process. A national survey conducted in 2003 confirms the lack of a structured screening program in the country, showing that 49.7% of women above the age of 50 have never been submitted to a mammography, and, among those who had done the exam, 18% did it more than 2 years ago [15].

### The Porto Alegre Breast Health Intervention Cohort

Considering the above, the Associação Hospitalar Moinhos de Vento, in a partnership with the City Health Department of Porto Alegre and a non-profit organization (Breast Institute of Rio Grande do Sul), initiated a prospective breast cancer screening study, denominated Porto Alegre Breast Health Intervention Cohort (NMPOA – Núcleo Mama Porto Alegre). The two primary objectives of this project are:

(1) To test the effectiveness of a centrally structured mammography screening and early treatment program, based on active women search, close contact with the BHU, promoting a multidisciplinary and humanized care for an underserved population from a developing country;

(2) To verify the profile of breast cancer risk factors in the sample studied, evaluating the frequency and possible contribution of established risk factors and attempting to identify new ones.

A secondary objective of the study is the estimation of the cost-effectiveness of such a program.

## Methods and design

### Study design, setting and participants

**Figure 1:**
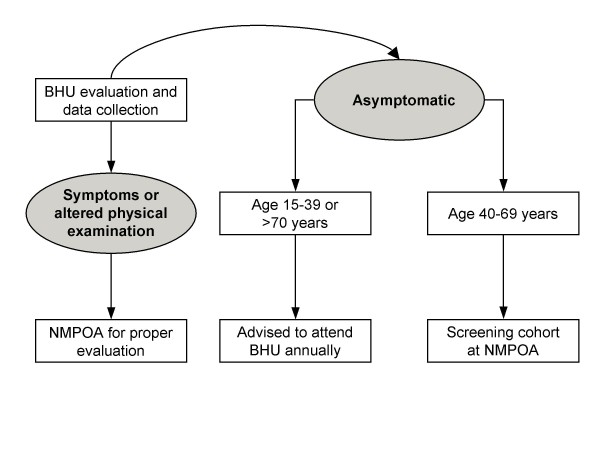
**Flowchart of patients in the study**. BHU: Basic Health Unit; NMPOA: Núcleo Mama Porto Alegre.

The NMPOA project is a breast cancer screening and early treatment program, with evaluation of risk factors for breast cancer in an intervention cohort. From April 2004 through March 2006, 9,218 women aged 15 years or older visiting one of the 19 participating BHUs from Porto Alegre were evaluated in a cross-sectional study, which included: (1) an entry form questionnaire regarding risk factors for breast cancer – including family history (FH) of cancer – and presence of breast symptoms, and (2) breast examination by a trained professional (nurse or medical doctor). All patients with breast complaints or an abnormal physical exam were referred to NMPOA for further evaluation. Women between 40 and 69 years of age were invited to participate in the screening cohort. Women without symptoms and outside the targeted age group were advised to attend the BHU once a year for physical examination. Figure 1 displays the evaluation flowchart. Women who participated in the cross-sectional study and complete 40 years of age during the project period, which was set to last until 2014, are also being invited to join the screening cohort.

The accrual was done at the participating BHUs both actively (i.e., lay health community workers inviting women in their own homes) and passively (women that attended the BHU for any reason being invited to the project). Although women from all ages above 15 could be enrolled in the cross-sectional study, the main emphasis was the invitation of women between the ages of 40 and 69 years, since that would be the group which would enter the screening cohort. The total number of women between 40 and 69 years old in the region was approximately 16,000, according to the most recent national census (year 2000), and the accrued number was about 4,000 in this age range.

The screening program is based on annual mammographies in women between the ages of 40 and 69 years, which are always preceded by physical examination performed by a breast surgeon or a trained nurse. One of the main features of the program is its close control of the frequency of visits, based on a computer system that generates monthly reports on the due dates of all participants. The close contact and continuous feedback to the BHUs professionals guarantees that this strategy is put into practice with maximum effort, in order to achieve optimal adherence to the screening program.

The proposal of the NMPOA project includes the performance of all clinical, imaging and pathology exams, as well as clinical visits and surgical treatment, in the same center. The health care approach is multi-disciplinary, including nurses, breast surgeons and a nutritionist. All mammographies are performed on the same day of the clinical visit, and they remain stored at the center. This integration of screening, diagnosis and treatment in the same center is an effort to shorten the time of delivery of appropriate treatment in each newly diagnosed breast cancer case.

All mammographic exams done at NMPOA are interpreted only by radiologists who are specialized in breast cancer and follow rigorous international standards of quality control. The radiologist classifies mammographies using density and risk criteria, purposed by the BI-RADS (Breast Imaging Report and Data System) classification [16]. Women with a mammogram classified as BI-RADS 3 are asked to return in six months for a new exam. If a mammogram is classified as BI-RADS 0, a complementary exam is necessary, and if the result is a BI-RADS 4 or 5 image, a biopsy is performed. Patients who have a diagnosis of breast cancer are operated in the same center.

The imaging screening consists of mammograms in patients older than 40 and ultrasound starting at an age 10 years younger than the relative's age when diagnosed with BC, not starting before the age of 25 (that is, if the relative was diagnosed before the age of 35, the patient would start ultrasound only when she was 25).

Participants of the cross-sectional study older than 18 years answering positively to at least one of the FH questions described below were referred to genetic cancer risk assessment. If the risk assessment yielded moderate or high genetic risk (estimated lifetime breast cancer risks greater than 20% or a family history suggestive of a hereditary breast cancer syndrome), the participant was invited to attend clinical visits and imaging screening at the NMPOA center, being followed by breast physicians and geneticists every six months. The main goals of the genetic evaluations are to determine: (1) the prevalence of a first-degree FH of cancer and characterize the type of cancer, age at diagnosis and relationship of the cancer-affected relative to the proband; (2) the lifetime breast cancer risk estimates in women attending the cohort and reporting a family history of breast and other cancers; (3) the prevalence of hereditary breast cancer phenotypes in a population-based sample of women in Latin America; (4) the contribution of germline mutations in known breast cancer predisposition genes among women from the cohort reporting a FH of cancer and fulfilling criteria for the clinical diagnosis of a breast cancer predisposition syndrome. Preliminary reports on the results of the genetic evaluation have been published elsewhere [17].

### Measurements and data collection

The questions regarding FH in the cross-sectional study focused on features that have been associated with an increased likelihood of clinically significant *BRCA *mutations, such as early-onset, bilateral or male breast cancer, multiple primary cancers of the breast and ovary, first-degree or multiple-case family history of breast or ovarian cancer. In addition, a question about FH of BC and/or colon cancer was included due to a previous suggestion of a higher than expect prevalence of such association in cancer genetic clinics of Porto Alegre.

Additional risk factors evaluated in the cross-sectional study included smoking, obesity, alcohol consumption and history of breast diseases. Current smokers were defined as those who smoked at least one cigarette per day; never smokers those who have never tried a cigarette in their life; and former smoker were defined as those who had stopped smoking for at least one year. Educational level of the participants (as a proxy of social status) was also inquired, and was measured by the years of schooling.

For the approximately 4,000 already registered women in the annual mammographic screening, a complete clinical form was filled when they joined the cohort, containing gynecological-obstetric data (such as number of pregnancies and hormone use), family history, medical history and physical exam, which includes anthropometric measurements and breast examination. Every time the participant returns for clinical evaluation and annual mammography, the answers are updated, since many of these risk factors are dynamic. BI-RADS data from all mammographies, including density score, are also stored. If a tumor is diagnosed, all data concerning staging, pathology report, hormone receptors and both surgical and oncological treatment are registered.

Participants who do not wish to continue in the screening program (or enter it, in the case of women reaching the age of 40) are requested to fill a form with the motive of withdrawal. This is also being recorded, in order to understand reasons for non-compliance, a major problem in Brazil [15,18-22].

For the patients who need complimentary investigation (other imaging modalities and biopsies), as well as in women who undergo surgical and oncological treatment for cancer, length between each step of care is been recorded. We intend to compare these data with other national registries in the PHS [23] in order to have a promptness and efficiency indicator of our model of care.

### Power calculations

The best parameter to show the effectiveness of our program would be breast cancer associated mortality. However, considering the very long breast cancer related death survival in asymptomatic women (especially in effective screening programs, where the detected cancers tend to have an excellent prognosis), and taking into account the well established relationship between tumor staging and survival [24], we chose this variable as the primary endpoint of our program. Since breast cancer survival has a significant drop even in stage II cases, we considered only staging equal or less than stage I as a favorable outcome to the screening program.

In search of an adequate comparison value, we considered that the staging of all breast cancers diagnosed during the study period in our city in a similar population (that is, in women from the same age group seen at public hospitals) would be suitable. These data are available upon request in the City Health Department. The total number of women between 40 and 69 years today in Porto Alegre is 247,000 [25], of which 75% (185,000) are estimated to have all their health care provided by the PHS [12]. We have already enrolled more than 4,000 women in the screening project, and, considering the expected number of women entering the cohort as they reach the age of 40 years, we estimate that we will have an average number of 4,250 women during the 10 year duration of the project. The correct number of the comparison group is therefore 180,750 women (185,000 minus 4,250), yielding a rate of circa 40 to 1 between the two samples. Applying the estimation of 119 breast cancer cases per 100,000 to our city in the current year [11], the number of cases in NMPOA would be 5 per year. Considering a 70% rate of tumor staging greater or equal to II in the city [9], it would be necessary to have 45 cases in the NMPOA cohort to show a 35% reduction in this rate, with an alfa = 0.05 and 90% power. We decided to achieve a 10% higher number of cases to overcome possible incorrect estimates in this calculation. Therefore, we decided to set the study period to ten years, in order to achieve 50 cancer cases in our cohort.

### Statistical analysis

The proportion of cases at or below stage I in the NMPOA cohort and the same proportion in the city will be compared by the Fisher's exact test. Continuous variables, like the time frames between the steps of care, will be compared with Student's t-test. We are also planning to perform a cost-effectiveness analysis of this intervention, in which a Markov model will be employed.

### Ethical issues

This project was approved by the ethical review board (ERB) of the City Health Department and of Associação Hospitalar Moinhos de Vento. The genetics project was also approved by the ERB from Hospital de Clínicas de Porto Alegre, where all biological material are stored. All subjects were provided a copy of the written consent before study entry and were assured of their anonymity and confidentiality of data collected.

### Characteristics of the study population at baseline

The screening cohort has already enrolled 4,037 women. Considering the number of women registered in the cross-sectional study who are reaching the age of 40 and will enter the cohort until the year 2014, this number will probably reach 4,500 until the end of the project.

Table 1 shows the baseline characteristics of the screening cohort. This population has a high average number of pregnancies (3 or more in over 60% of women), a low percentage of late menopause and low rate of hormone replacement therapy use. Women in our cohort have their first delivery at a young age (21.4 ± 5.1 years). The presence of relatives with any type of cancer is high (33.7%), and the prevalence of overweight and obesity is also elevated (73.7%). Education level is low in this population, as can be seen by the high number (56.5%) of people who haven't completed Primary School (Brazilian equivalent to elementary and middle school) and the considerable illiteracy rate (6.5%).

**Table 1:** Baseline cohort characteristics

Variable	N	(%)
Age (yrs)*	50.6	± 7.7
Race		
White	2,964	73.4%
Black	700	17.3%
Other	373	9.3%
Number of pregnancies		
0	322	8.0%
1–2	1,037	25.7%
3–4	1,349	33.4%
>4	1,329	32.9%
Education level		
Illiterate	259	6.5%
Between 1 and 7 years of schooling	2.238	56.5%
Between 8 and 11 years of schooling	1.380	34.9%
College degree	83	2.1%
Body Mass Index		
<25	1,062	26.3%
25–30	1,502	37.2%
30–40	1,320	32.7%
>40	153	3.8%
Age at first delivery (yrs)*	21.4	± 5.1
Menopause after 55 yrs old†	88	4.4%
Menarche before 12 yrs old	872	21.6%
History of HRT use	485	12.0%
History of OC use	3,331	82.5%
Smoking status		
Current smoker	1,170	29.0%
Former smoker	393	9.7%
No historry	2,474	61.3%
First-degree relative with history of cancer (any site)	1,395	33.7%
First-degree relative with history of breast or ovary cancer	248	6.1%
Previous breast biopsy	154	3.8%

HRT = Hormone Replacement Therapy, OC = Oral Contraceptives

* expressed as mean ± standart deviation; † percentage over 2,002 women who already entered menopause

There were 1286 women who responded positively to one of the high genetic risk questions in the cross-sectional study, 902 of whom were further referred to genetic cancer risk assessment sessions. Of these, 214 had a pedigree suggestive of a hereditary breast cancer predisposition syndrome. Preliminary results from the genetic evaluation have been published elsewhere [17].

Incidence of cancer so far has been 117 cases per 100,000 person-years (9 cases over 7,656 person-years), a number very similar to the number of cases predicted in the city for the year 2008, 119 cases per 100,000 women. Compliance to the annual screening has been approximately 60% so far, and the rate of participants who had their last mammogram not more than 2 years ago is almost 80% [25] (a more detailed discussion of the adherence to the program will be published shortly).

## Discussion

In this article, we describe the main characteristics of the NMPOA project, which was designed to test a model of breast cancer screening and early treatment in an underserved population of a developing country. Also, we aim to evaluate the prevalence of risk factors for breast cancer in this population, which is a representative sample of our city and presents a high breast cancer incidence. The first important finding of the study was the low rate of patient inclusion in the cross-sectional study, especially in the age group which was the target of the screening cohort (25% of eligible women in the region). Although we cannot be sure about the total number of women reached by the active and passive search, this number suggests a low adherence to preventive health care in this population. This is in accordance with national data, which showed that almost 50% of women above 50 years old have never done mammography, and almost 10% had done their last exam three or more years before [12].

The incidence of breast cancer in this population is high, being comparable to developed countries and largely exceeding the Brazilian average incidence of 51 cases per 100,000. The cancer rate becomes quite intriguing when we confront it with the baseline data of our cohort: the majority of breast cancer risk factors in this population have a low prevalence (such as low hormone replacement therapy use, low number of pregnancies and a young age at first delivery), fostering the idea that unknown risk factors might be playing a major role in this region and reinforcing the importance of understanding the distribution of breast cancer risk factors in different populations. The incidence of cancer observed so far in the screening cohort suggests that this sample is representative of our city.

Our longitudinal collection of risk factor data, especially the genetic risk factors, is very important for an accurate measurement, since this factor can change over time, as new information about cancer development in relatives of the patient becomes acknowledged. The design and sample size of our study will probably be sufficient to respond the primary question of our study, regarding the effectiveness of the model. However, we recognize that the correlation of risk factors and breast cancer will probably be underpowered. Nevertheless, we believe that our study can contribute with others in the field, and, in a pooled analysis through meta-analytic techniques, help us understand the high incidence of the disease in a large area in our country.

We consider that the high rate of compliance so far is one the main strengths of your study. This is achieved by patient education, with information about the importance of the screening program, and intense contact with the BHUs, with provision of lists of patients who should come next month. We also supply transportation for women with very low financial resources, what helps to keep attendance. Every three or four months, we select a region with important problems of attendance and provide a special bus to take them to the PABHC.

This project aims to establish a model of breast health attention directed to underserved women that rely only on health care provided by the public sector. The prompt access of women in our study to the reference service, as well as the clinical exam done by a specialized professional and high quality mammography are fundamental issues for early diagnosis of breast cancer. This is a major step to reduce breast cancer mortality worldwide.

## Abbreviations

**ASM**: Age-standardized mortality; **BI-RADS**: Breast Imaging Report and Data System; **BC**: Breast cancer; **BHU**: Basic healthcare unit; **ERB**: Ethical review board; **FH**: Family history; **HRT**: Hormone replacement therapy; **OC**: Oral contraceptives; **NMPOA**: Núcleo Mama Porto Alegre (Porto Alegre Breast Health Intervention Cohort); **PHS**: Public Health System.

## Competing interests

The authors declare that they have no competing interests.

## Authors' contributions

MC was responsible for study design and manuscript review, and also participated in the manuscript drafting. RR was responsible for manuscript drafting and data analysis. BW, PAP, DD, PK, LF, JF, MG and PP participated in the study design. AB, PAP, JZ, JG and GS participated in data collection and were involved in patients' care. All authors read and approved the final manuscript.

## Pre-publication history

The pre-publication history for this paper can be accessed here:


